# Towards the Sustainability of English Language Teachers Professionalism *via* Professional Development Programs: Extrinsic and Intrinsic Satisfactions

**DOI:** 10.3389/fpsyg.2022.828060

**Published:** 2022-05-27

**Authors:** Ruzana Omar, Radzuwan Ab Rashid, Sarah Yusoff, Hanita Hanim Ismail, Hadeel Saed, Baderaddin Yassin, Omar Ali Al-Smadi

**Affiliations:** ^1^Universiti Teknologi MARA, Academy of Language Studies, Shah Alam, Malaysia; ^2^Faculty of Languages and Communication, Universiti Sultan Zainal Abidin, Kuala Terengganu, Malaysia; ^3^Faculty of Computer and Mathematical Sciences, Universiti Teknologi MARA, Shah Alam, Malaysia; ^4^Faculty of Education, National University of Malaysia, Bangi, Malaysia; ^5^Faculty of Arts and Humanities, Applied Science Private University, Amman, Jordan; ^6^Language Center, Al-Ghad International Colleges for Applied Medical Sciences, Riyadh, Saudi Arabia; ^7^Applied College, University of Ha'il, Hail, Saudi Arabia

**Keywords:** intrinsic satisfaction, extrinsic satisfaction, teacher professional development, ESL, motivation, primary school teacher

## Abstract

With a great emphasis on understanding teacher satisfaction in designing and improving professional development programs (PDPs), this study investigated the intrinsic and extrinsic aspects of satisfaction among Malaysian English as a Second Language (ESL) primary school teachers after finishing their first-degree program. A mixed-methods design was employed by using both quantitative methods (by administering survey research) and qualitative methods (through the use of items of the semi-structured questionnaire). The survey questionnaire was adapted from OECD Teaching and Learning International Survey (TALIS) questionnaire to measure the level of intrinsic and extrinsic teacher satisfaction among the samples. Descriptive analysis was used to analyse the survey research data collected from 30 secondary school English language teachers in Kuala Terengganu, east coast of Malaysia. The findings revealed that personal gains did not contribute much to PD activities in relation to the level of the teacher's extrinsic satisfaction after following their first-degree PD program. The study indicates that teachers differ from each other in terms of the source of their motivation for PD, whether intrinsic or extrinsic, and the type of development they aim for. This is hoped to show direction to the policymakers and organizers of PD programs in enhancing the program by taking into consideration of the teacher satisfaction, seniority, and professional background.

## Introduction

The quality of professional development program (PDP) influences teacher characteristics of their satisfactions. Teacher satisfaction is achieved once they have fulfilled the needs of their expectations, in terms of the quality of lecturers, the intrinsic and extrinsic needs that had been met, input that they have received, and the new knowledge and skills that they had gained, specifically in the field of teaching and language skills. Hence, it is imperative for educational institutions to identify the teacher satisfaction with their PD to provide quality teacher PDPs in the future.

Due to this fact, the Education Ministry of Malaysia established a committee in 1995 to examine teachers' PD in Malaysia in order to increase the teaching profession (Jamil et al., 2011; Rashid et al., 2017). Based on the recommendations given by the committee, teachers were highly encouraged to attend in-service courses and further their education. Teacher centers were thus entrusted with the task of improving teaching skills in a professional way. After the formal establishment of these teacher centers, various activities were designed at improving the quality of teachers. Many teachers had registered themselves to develop their professionalism in the program.

In relation to teacher's PD, their involvement in PDP gained satisfaction due to internal or external motivation. Externally motivated individuals are driven by incentives, such as an academic degree, salary raise, and improvement in the opportunity for career development (Ryan and Deci, 2000; Ab Rashid, 2018). In contrast, internally motivated individuals, who are driven by interest and curiosity, believe that this is the right way to act and enjoy as a result of participation in the PD activity. Understanding the correlation between the sources of motivation for associating PD with teacher satisfaction can direct policymakers in seeking effective ways of encouraging in-service teachers to engage in PD activities. This study attempts to examine teachers' intrinsic and extrinsic satisfaction after their involvement in the primary school after completing their first-degree program. The quality of the program will be determined by the level of the teacher satisfaction. It would also be interesting to investigate the correlation between the teacher's intrinsic and extrinsic satisfaction with demographic profile after involving in the PDP.

## Teacher Satisfaction and Professional Development

Teachers' level of satisfaction in PDPs determines the degree of their perceived control on different aspects of PDP, such as determining school-based or non-school-based programs, identifying their needs in developing the instruction programs, and having the freedom to decide participation in the program. Based on the theoretical background, teachers' inclination to participate in PDPs is also determined based on their teaching jobs, such as level of job involvement, and administrative support as well as the latter's encouragement (Nir and Bogler, 2008; Had and Rashid, 2019). This is even more surprising when 38% of teachers declare that they do not have any autonomy nor influence over their target in PD (Worth and Van den Brande, 2020). Hence, there is a vast possibility of improving the rate of satisfaction and retention among teachers in increasing teacher agency with regard to their PD targets.

Teacher satisfaction is also due to the influence of the work environment where fulfilling psychological needs may cause teachers to contribute and thrive effectively. In fact, it improves the quality of schools as a work environment where trust issues between the supervisor and the teacher could be reduced due to the latter's willingness to perform beyond institutional requirements, resulting from the latter's satisfaction. To be more precise, there is a relationship between greater teacher control and an increased level of satisfaction with PD processes (Ryan and Deci, 2000; Had and Rashid, 2019).

There are different constructs that specify job satisfaction as identified by some theorists. Generally, the components are divided into two groups, namely, internal (or intrinsic) and external (or extrinsic) factors (Ewen, 1964). Internal factors comprise different motivating aspects (e.g., work, work achievement, promotion and recognition, and responsibility) that promote job satisfaction. In contrast, external factors (e.g., company policy and work security, working conditions, employees' status and salary, and collegial relationships) are essential to prevent job dissatisfaction, which thus create short-term satisfaction. However, these factors do not lead to long-term satisfaction and motivation.

The PD, salary increase, and work conditions are important to inculcate teachers' external motivation (Rautakivi et al., 2019). In contrast, internally motivated individuals who are driven by interest and curiosity believe that these two aspects govern the right attitude while enjoying teaching. Understanding the relationship between PD motivation and satisfaction can direct policymakers to seek effective ways at encouraging in-service teachers to be engaged in PD activities. Following this line of thought, increasing teacher motivation has an influence on job satisfaction, which is observed to be a critical variable in life. For example, teacher satisfaction is often identified in terms of teacher quality, retention, and organizational commitment in school areas (e.g., academic achievement, student behavior, student satisfaction, teacher turnover, and management performance) (Mathews-Aydinli, 2008). The third set of variables associated with PDP satisfaction is the program characteristics. The program elements should meet the needs and expectations of the participants so that they are satisfied. Relevant elements, including objectives, the cultural target group (i.e., focus on teaching strategies vs. subject matter) and the education level to which it aims (Nir and Bogler, 2008).

Ewen (1964) directed future research to look into other characteristics that affect satisfaction, thus appropriating the purpose of this study (i.e., to identify the characteristics of teacher satisfaction with their PD program). It would also be interesting to associate teacher satisfaction with their needs as primary ESL teachers. From the finding of the study, relevant attributes of teachers' potential needs had been discovered. Hence, the findings would attend to teachers' needs, besides relating to factors that concern teachers' continuing PD. In the case of this study, the participants are perceived to be driven to participate in continuing their education due to a steadily increasing number of graduated in-service teachers teaching in primary schools which had caused the more experienced teachers to feel intimidated. This has become a driving factor for the need to upgrade themselves. Attractive incentives by the government in terms of salary and grade increase as well as subsidies in terms of tuition fees have also drawn primary school ESL teachers to further their studies.

## Motivation in Relation to Intrinsic Satisfaction of Professional Development Program

Intrinsic satisfaction is associated with a person's internal motivation. Hennessey et al. (2015) explained that intrinsic motivation is people's enthusiasm to do beyond their interests. Hence, organizations need to raise awareness on celebrating teachers' intrinsic motivation. Instead of avoiding punishment or promising rewards, the latter would strive for challenges or even pursue happiness. Intrinsic motivation is also attributed to teachers' desire to develop professionally and consider this development to be part of their personal responsibility as teachers—a commitment they made to themselves and to the profession (Avidov-Ungar, 2016). Another description of teachers' focus on intrinsic motivations for PD includes their sense of satisfaction and fulfillment from teaching challenges.

People, who are internally motivated, are more inspired in their PD activities and job performance (Cherry, 2014). In the workplace, work efficacy and performance observably increase due to extrinsic motivation (e.g., remuneration and job recognition). Yet, actual work performance and its quality are influenced by intrinsic factors. This study, thus, aims to investigate teachers' intrinsic and extrinsic satisfaction based on the variable, i.e., primary school teachers' first-degree program. It is believed that the teachers' driving factors to further their studies are due to the extrinsic factors, namely, salary increment and raise in teaching grade. The results reveal that the perceived level of extrinsic satisfaction that they had gained is based on data collection. The scale of the level of satisfaction used the Likert-type scale measurement. The level of intrinsic satisfaction, namely, self-efficacy, superior, and communal recognition, had been recorded. Therefore, whether it is teachers' extrinsic or intrinsic motivation, it roots from within. The measurement of the level of intrinsic and extrinsic satisfaction after attending a part-time first-degree program among the selected primary school teachers would serve as an eye-opener to stakeholders on teachers' perceptions about their PD and would provide essential suggestions for improvement and necessity for the development of teachers' PD courses or programs.

## Motivation in Relation to Extrinsic Satisfaction of Professional Development

Extrinsic satisfaction is the level of satisfaction attained from externally driven motivation. Extrinsic motivation refers to any activity that includes elements of stress, uncertainty, or apprehension with a main purpose of pursuing a desired object (Lindenberg, 2001). Extrinsic motivation is relevant to external influences, such as recognition, rewards, and promotion. While motivation can be increased by offering rewards in some cases, it must be done responsibly since Cherry (2014) found that over-recognition (e.g., appreciation) can reduce teachers' motivation. The need for positive recognition, the desire to emulate a specific role model, and the hope of an offered promotion are regarded as extrinsic motivation (Avidov-Ungar, 2016). In fact, Bouwma-Gearhart (2012) argued that teachers are motivated to engage in teaching PD due to extrinsic motivations. The results present a challenge to any research organization which has determined that the faculty should be intrinsically motivated to be involved in PD.

In another instance, Rautakivi et al. (2019) revealed that the teaching profession in Cambodia does not offer a rewarding career as a form of substantial intrinsic motivation, which is much needed by local teachers due to their extrinsic motivations (e.g., social values, conducive working conditions, and career incentives), which is hard to be found. Hence, these teachers need to rediscover their initial purpose of becoming teachers. Hence, their perception toward extrinsic satisfaction in the form of tangible rewards (e.g., career incentives, better working conditions, and social values) would change and result in a balanced satisfaction of both intrinsic and extrinsic.

## Materials and Methods

### Instrument and Procedure of the Study

A mixed-methods design was adopted by utilizing both quantitative methods (by using survey research) and qualitative methods (by using items of the semi-structured questionnaire). Triangulation was used to strengthen the data collected.

The first instrument to collect data for this study is a survey questionnaire, which was adapted from the OECD Teaching and Learning International Survey (TALIS) questionnaire to measure the level of intrinsic and extrinsic satisfaction of the samplings. Five-point Likert-type scale items were used to examine the level of satisfaction among the participants after undergoing the program. Questionnaires were used to gather data on teachers' self-satisfaction after participating in PD activities. The questionnaire is divided into three section, namely Part 1 (demographic data), Part 2 (what are the intrinsic satisfactions on English language teachers after completing a degree for PD on a part-time basis?), and Part 3 (what are the intrinsic satisfactions on English language teachers after completing a degree for PD on a part-time basis?).

The second instrument that answers the second research question is a semi-structured interview, which was used to strengthen obtained data from the survey questionnaire. The 10–15 min interview was conducted with purposefully selected participants. The interviews were carried out in each school on a day that was convenient to the participants. A room was chosen where no interruptions would hinder the audio-tapping of the discussion. A digital audio recording device was used to record the interviews. Six semi-structured questions were constructed to examine teacher satisfaction on:

i) knowledge and skills obtained,
ii) quality of trainers/lecturers,
iii) qualification of trainers/lecturers.

According to Djamba and Neuman (2002), flexible and creative approaches to qualitative data analysis are highly desirable, since words are frequently imprecise, context-dependent, and usually suggest more than one interpretation; this is one of the reasons for not opting to use software analytical tool for the second part. Furthermore, such tools have proven to be a disadvantage to capture the meaning of paralinguistic features, such as laughter, silences, hesitations, and stress variations, which in many occasions may either undermine or contradict the verbal messages that participants appear to be saying.

### Participants

**Figure 1:**
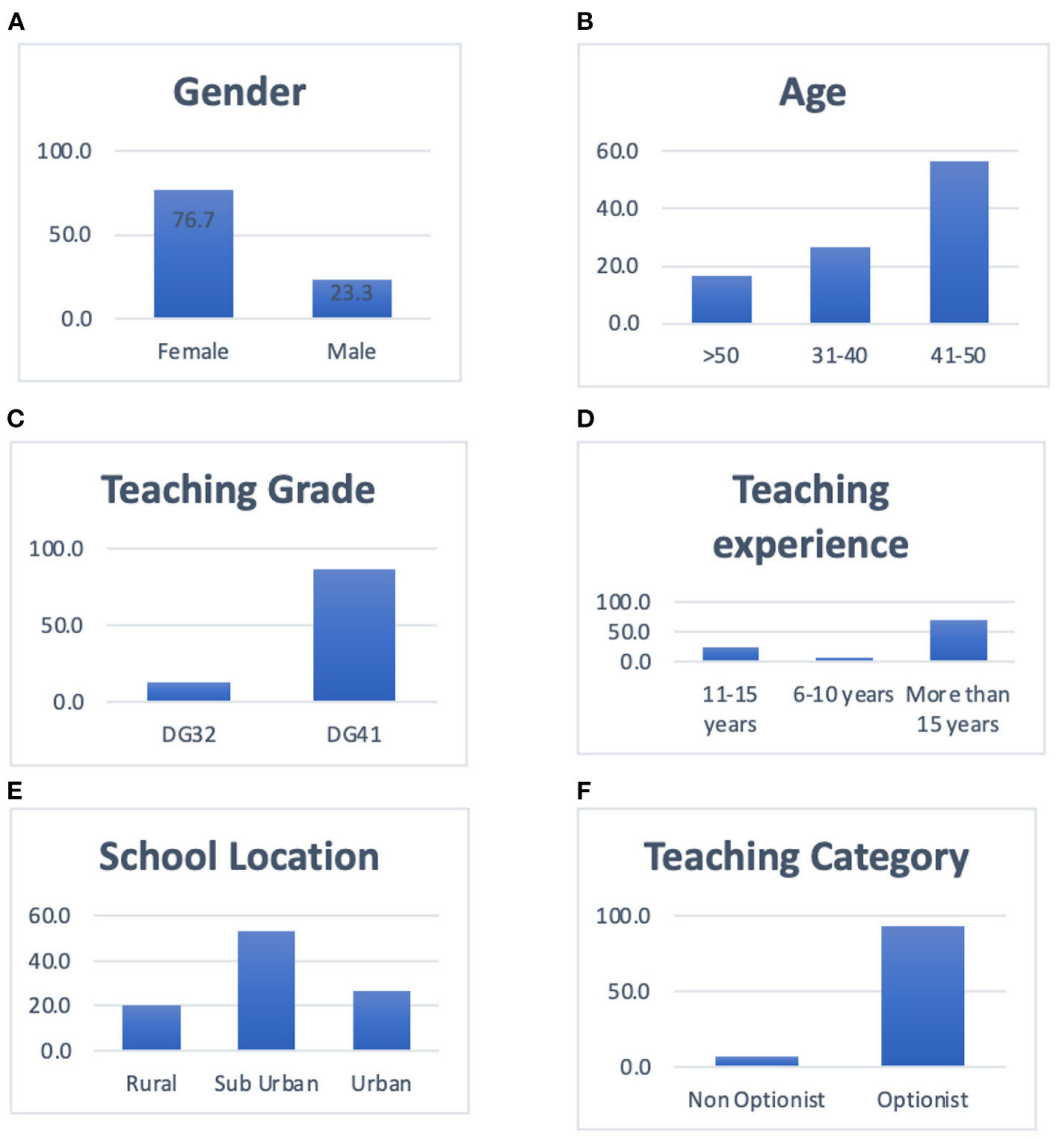
Bar chart for demographic profile. **(A)** Bar chart for gender. **(B)** Bar chart for age. **(C)** Bar chart for teaching grade. **(D)** Bar chart for teaching experience. **(E)** Bar chart for school location. **(F)** Bar chart for teaching category.

A total of 30 in-service English language primary school teachers who had undergone PD and obtained their first degree on a part-time basis were selected. The primary reason for choosing participants with this profile is their provision of richer accounts of the impact that these professional life experiences have on their satisfaction. From the thirty participants, six teachers were chosen for a semi-structured interview. They were chosen based on the diversity of their experiences that further contribute to the richness of the study. It also enables a greater understanding of these teachers' satisfaction with the first-degree program in terms of the quality of the program and the quality of the lecturers, and their qualifications. The six chosen participants of this study have diversity in terms of their age, gender, school location, responsibilities in schools, and teaching experiences. The different backgrounds and varied experiences of the participants had contributed to rich data generation. Refer to Figure 1 for detailed information of the participants.

### Data Analysis

Data obtained from the questionnaire were analyzed using SPSS. The items in the questionnaires required the respondents to indicate responses based on a 5-point Likert-type scale. In this study, participants with a score of 4.0 and above (agree to strongly agree) are considered positive responses, while those with a score of 3.9 and below (disagree to strongly disagree) are considered negative responses. Descriptive analysis was used to analyze the data. The findings obtained are reported in two parts. Part 1 presents the data collected regarding the level of extrinsic satisfaction gained by the ESL teachers after following the first-degree program on a part-time basis. Part 2 shows the intrinsic self-satisfaction of ESL teachers after following the program. In addition, this study also conducted independent samples *t*-test and one-way ANOVA to identify any mean difference between groups in the demographic profile.

Following that, Interview Follow-Up Questionnaire (IFUQ) was conducted to further understand the level of the teacher satisfaction after following the program. In this stage, their level of satisfaction about the overall program regarding the academic's quality of teaching, satisfaction on knowledge gained from PDP, as well as their appraisal of the competency of the trainers' and lecturers' teaching the program and the lecturers' level of experience were also gauged.

## Results

### Results of TPD Satisfactions From Survey Questionnaire

Table 1 indicates results on the mean and standard deviation of teachers' extrinsic level of satisfaction after involving in the first-degree program.

**Table 1:** Frequency table for demographic profile.

**Variable**	**Category**	**Number of subject (*n* = 30)**	**Percentage (%)**	**Graph**
Age	>50	5	16.7	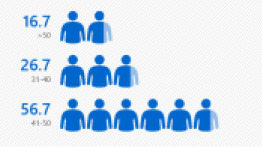
	31–40	8	26.7	
	41–50	17	56.7	
Gender	Female	23	76.7	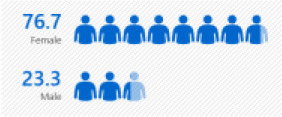
	Male	7	23.3	
Teaching grade	DG32	4	13.3	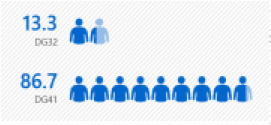
	DG41	26	86.7	
Teaching experience	11–15 years	7	23.3	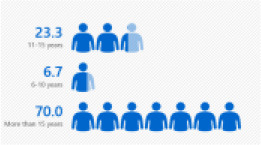
	6–10 years	2	6.7	
	More than 15 years	21	70.0	
School location	Rural	6	20.0	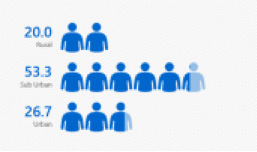
	Sub urban	16	53.3	
	Urban	8	26.7	
Teaching category	Non-option	2	6.7	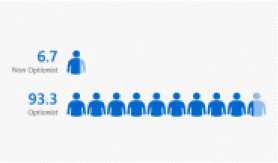
	Option	28	93.3	

The findings obtained from the demographic data are presented in Table 1. The findings revealed that the percentage of the 7 (23.3%) male and 23 (76.7%) female respondents was not equally distributed where the percentage of female respondents outnumbered the male respondents. This is partially due to the bigger number of female teachers' teaching in primary schools in Kuala Terengganu. The critical shortage of male teachers in Kuala Terengganu also applies to other states in Peninsular Malaysia, where it is difficult to attract male students into the teaching profession, especially in the teaching of the English language. A newspaper article reported that men were not interested in becoming teachers, which brought about a concern for the Ministry of Education (MOE) since the ratio of male to female teachers was 20:80. Due to that reason, it was reported that vigilant efforts by the ministry are made to attract more men to become teachers, such as providing them with incentives or lengthening the training period.

Results also indicated that 100% of the respondents were above 30 years, in which 26.7% of the respondents were in the 31–40 age category, 56.7% in the 41–50 age category, and another 16.7% in the above 50 age category. Hence, the participants represent the most mature and senior teachers in their schools where the majority is in the range of 41–50 representing 56.7% of the overall participants in the study, followed by teachers of the age bracket of above 30 years (26.7%) and above 50 categories (16.7%), respectively. Teaching experience was another variable explored in this study. Based on the results displayed in Table 1, 23.3% of the teachers had between 11 and 15 years of teaching experience, while 21% of the teachers had above 15 years of teaching experience. The results also showed that younger teachers with 6–10 years of teaching experience recorded merely 2 (6.7%) respondents. This shows that these participants are highly experienced in the teaching of the English language in a primary school based on the range of their service that would contribute to the richness of the data in identifying the level of teacher satisfaction after following the PDP.

Table 1 shows that the majority of teachers teaching in suburban schools were 16 (53.3%) respondents as compared to those teaching in urban schools, which was the second-highest number, both eight (26.7%) and six teachers (20%) teaching in a rural school, respectively. Most participants are English teachers teaching in suburban areas due to the fact that the MOE took into consideration that the geographical location of the schools ought to be the nearest to the location of the designated campus chosen for their first-degree program.

The findings obtained also revealed that all the respondents opted for English as their specialization (91.6%) as compared to those who did not opt for English as their specialization (8.3%). Since primary schools in Malaysia are still seriously lacking proficiency amongst English language teachers, the increasing number of English-option teachers teaching in the schools is very encouraging.

### Reliability Test

The Cronbach's alpha coefficient is the most widely used internal consistency measure. When using Likert-type scales, the test is regarded as the most acceptable measure of reliability. A total of 13 items from the two constructs in the study, i.e., extrinsic and intrinsic satisfactions, were designated for the initial instrument to investigate the intrinsic and extrinsic aspects of satisfaction among ESL primary school teachers after following the first-degree program. The reliabilities of both parameters were high (all at or above Cronbach's alpha ≥ 0.908). The reliability coefficient (Cronbach's alpha) for extrinsic and intrinsic satisfactions was 0.908 and 0.935, respectively, indicating that the items in the instrument were highly accurate internally. The Cronbach's alpha coefficient for each construct is shown in Table 2. The result suggests that the internal consistency of the teacher satisfaction scale is high.

**Table 2:** Reliability analysis.

**Constructs**	**Number of items**	**Cronbach's alpha**
Teacher's extrinsic satisfactions	7	0.908
Teacher's intrinsic satisfactions	6	0.935

### Descriptive Statistics

Based on the results shown in Table 3, Item number 7, which is “More networking with teachers of other schools”, has contributed to the most satisfaction rate after following the first-degree program, followed by Item number 5 (Gateway to further education) as the second level of satisfaction gained by the teachers from involving in the first-degree program which recorded the mean of 3.93 and standard deviations of 0.842, 3.73, and 0.691, respectively. Item number 5 (Public recognition from the principal) charted the third-highest (M = 3.63, SD = 0.718). More opportunities for PD courses, job promotion, salary increment, and a change in profession for career advancement are placed fourth, fifth, sixth, and seventh, respectively.

**Table 3:** Descriptive analysis for the level of teacher's extrinsic satisfactions.

	**Extrinsic satisfactions**	**Min**	**Max**	**Mean**	**Mode**	**Std deviation**
1.	Salary increment	1	5	3.40	3	0.724
2.	Job promotion	1	5	3.43	3	0.728
3.	More opportunities for professional development courses	2	5	3.53	3	0.681
4.	A change in profession for career advancement	2	5	3.33	3	0.758
5.	Public recognition from the principal/your colleagues	3	5	3.63	3	0.718
6.	Gateway to further education	2	5	3.73	4	0.691
7.	More networking with teachers at other schools	1	5	3.93	4	0.842

As shown in Table 4, Item numbers 1 (*Confidence in teaching skills*), 4 (*Improving interactions with students*), and 6 (*New self-identity*) have contributed to the most intrinsic satisfaction after following the first-degree program, which recorded the mean of 3.97, followed by Item number 5, which was “Self-efficacy” as the second level of intrinsic satisfaction gained by the teachers which recorded the mean of 3.87 and standard deviation of 0.681, respectively. Item number 2 (*Recognition from family members or communities*) charted the third-highest (M = 3.83, SD = 0.648). Finally, Item number 3 (*Confidence in English language communication*) recorded the last position with the mean of 3.70 and standard deviation of 0.750.

**Table 4:** Descriptive analysis for the level of teacher's intrinsic satisfactions.

	**Intrinsic satisfactions**	**Min**	**Max**	**Mean**	**Mode**	**Std. deviation**
1.	Confidence in teaching skills	3	5	3.97	4	0.669
2.	Recognition from the family/the community	3	5	3.83	4	0.648
3.	Confidence in English language communication	3	5	3.70	3	0.750
4.	Improving interactions with students	3	5	3.97	4	0.615
5.	Self-efficacy (belief in innate ability to achieve a goal)	3	5	3.87	4	0.681
6.	New self-identity	3	5	3.97	4	0.615

Table 5 shows that there exists a significance difference (with significance value <0.05) in the mean of the extrinsic satisfactions between teaching grade only. Other demographic profile has no significance as can be seen in Table 6. Teachers with grade DG32 have a higher mean satisfaction score (4.1071) than those teachers with grade DG41 (3.4872), as shown in Table 5. To sum up, extrinsic satisfaction for teachers with grade DG32 is better than those of grade DG41. Table 7 shows the mean difference between DG32 and DG41 in the extrinsic satisfactions.

**Table 5:** Mean difference for extrinsic satisfactions with demographic profile.

**Analysis**	**Category variable**	**Significance value for extrinsic satisfactions**	**Conclusion**
Independent *t*-test	Gender	0.215	Not significant
	Teaching category	0.997	Not significant
	Teaching grade	0.047*	Significant
ANOVA test	Age	0.510	Not significant
	Teaching experience	0.452	Not significant
	School location	0.129	Not significant

**Table 6:** Mean difference for intrinsic satisfactions with demographic profile.

**Analysis**	**Category variable**	**Significance value for intrinsic satisfactions**	**Conclusion**
Independent *t*-test	Gender	0.913	Not significant
	Teaching category	0.244	Not significant
	Teaching grade	0.378	Not significant
ANOVA test	Age	0.425	Not significant
	Teaching experience	0.079	Not significant
	School location	0.195	Not significant

**Table 7:** Mean difference for extrinsic satisfactions.

	**Teaching grade**	***N***	**Mean**	**Standard deviation**	**Standard error mean**
Extrinsic	DG32	4	4.1250	1.01265	0.50633
Satisfactions	DG41	26	3.8462	0.50315	0.09868

### Interview Follow-Up Questionnaire

The following section reports the findings obtained from the semi-structured interview. It reveals the findings of interview-follow up questionnaire (IFUQ) on teachers' level of satisfaction on the quality of the lecturers and the program.

#### Satisfaction of Teachers on Knowledge and Skills Obtained

Are you satisfied with the knowledge and skills obtained through the degree that you have completed?

##### Extract 1

Very satisfied. The knowledge and skills are highly relevant and applicable to my teaching. I personally, loves to learn linguistics, the part of how the phrase, the word, the sentence work together. From there, I get to understand how to structure the correct sentences, that's wonderful to help me in teaching language in the classroom. (P1/Q1/IFUQ).

The education courses offered by a university have to be relevant and practical for teachers to apply in schools for the benefit of their students. Participant 1 (P1) expressed his satisfaction toward the knowledge and skills that he had collected from the program, where he cited the knowledge and skills he had gained are highly relevant and applicable to his teaching. Based on the interview conducted, the lecturers have generally provided a satisfactory level in terms of the knowledge and skills for the teachers to apply after following the program.

##### Extract 2

Yes. All is good to me. I have been there for four years. Within that time, there was always new things that I had learnt in each semester. I remember that I was always looking forward to the new semester as I felt that each semester opens up the window to what I did not know about education, like technology in teaching where I've been taught about teaching aids, pedagogy, grammar teaching, to name a few.

##### (P2/Q1/IFUQ)

Based on the feedback given by Participant 2 (P2) as shown above, she seems excited to be a part of the program. The knowledge that she had received seemed to benefit her in the field of education. The area that she mentioned that she had learned in the program seems comprehensive and relevant to language teaching. It is essential for a learner to be interested and know what she had received in their PD education program so to enable better contribution and knowledge enhancement. Hopefully, this obtained knowledge would further be beneficial and applicable to her teaching in the classroom.

#### Quality of Trainers and Lecturers

How do you find the trainers/lecturers who trained/taught you throughout the degree?

##### Extract 1

The lecturers were very understanding and friendly. I was very happy with them. They're very competent, experienced and knowledgeable. I think they're also very active in carrying out researches and studies.

##### (P3/Q2/IFUQ)

The quality of trainers or lecturers in one institution plays a vital role in ensuring the production of holistic students who excel academically and non-academically. It is discernible that Participant 3 (P3) expressed his satisfaction toward the nature of the lecturer's code of conduct and maturity in handling matters in the institution while following the program. The lecturers are also observed to be competent, experienced, and knowledgeable, besides being prolific in carrying out their studies.

##### Extract 2

Approachable. I can discuss things with them. Maybe they treat us in such way, since we are adult learners, hahaha01_fmed-09-892205 (grinned). But, somehow it is their professionalism that causing me to respect them more. They treat us as a friend, but at the same time they were firm when it comes to the execution of assignments and tasks given to us during the lecture.

##### (P4/Q2/IFUQ)

The quality of the lecturer is the essence of the institution. An educator needs to know how to effectively teach adult learners. They should not be taught the same way as teaching fresh graduates. As adult learners are usually experienced teachers, the lecturers should have an open discussion, sharing their knowledge during the lesson rather than applying the spoon-feeding approach. The questions given in the form of open-ended are likely to be more suitable for adult learners.

#### Qualification of Trainers and Lecturers

Are you happy with their qualification/experience and competency? Why?

##### Extract 1

Yes. They are qualified, based on their knowledge and skill that I observed. Besides, the university must be strict in hiring their staff. I also believed that their experience must be abundance since they are teaching in a well-known local university. I am also not questioning their teaching capabilities since they seem to manage well. Maybe got one or two situations where I am not really happy with, especially during consultation where they tend to always change their opinions on things they asked us to do.

##### (P5/Q3/IFUQ)

UiTM is a public university in Malaysia where the requirement for a qualified lecturer is given serious consideration, where the hiring of lecturers is based on merit. As such, Participant 5 (P5) was satisfied with the qualifications of the lecturers due to that nature. Based on the interview finding, the majority of the participants were satisfied with the program and the quality of the lecturers. They agreed that the program has successfully produced many graduates among adult learners consisting of ESL primary school teachers.

##### Extract 2

I would say yes for many of them, but there were a few that I noticed not up to the par, though, I think (pause). For instance, I noticed that a few old generation of the lecturer are not very skillful on using the technology in teaching, like they would prefer to use old method when giving notes. I mean they don't like it if we were to present our mock teaching by recording and submit it to her online. Well, somehow maybe that wasn't allowed, hihi (cheeky).

##### (P6/Q3/IFUQ)

Based on the perspectives of Participant 6 (P6) shown above, she seemed to be generally satisfied with the qualification of the lecturers. Nevertheless, there were instances where it was noticeable with senior lecturers who lack technological skills. Educators need to upgrade their knowledge and skill in technology by attending courses and getting involved in PD to enhance their teaching, besides preparing themselves to be more technologically skilful lecturers. With the help of computers and Internet, e-learning brings changes to pedagogical strategies and ultimately improves the efficiency of teaching and learning (Omar et al., 2018).

## Discussion

From the data analysis on extrinsic satisfaction, it was found that personal gains did not contribute much to the teachers after they had completed their PDP. This contradicts the finding found in a study performed by Nasser and Shabti (2010) where externally motivated persons are driven by incentives, such as securing a university degree and getting increased salaries. Such a contradiction is due to the type of participants involved in the study, who are mature teachers. Based on the number of years and teaching experience in schools, the participants consist of senior primary school ESL teachers, with salaries almost reaching the “ceiling” of the salary scale. Hence, even with completing their first degree, the increment might not be much of a privilege or a difference to them. That contributes to the less feeling of extrinsic satisfaction among the teachers. The same factor was reasoned out to the “job promotion”, recorded at fourth place, where many of them held important posts and positions in their school due to their many years in service and experience. Thus, even with the new promotion exercise after completing their studies, it would not benefit them as they had already held good positions at their workplace, recorded at fourth placement. What Ryan and Deci (2000) argue that externally motivated individuals are driven by incentives, such as an academic degree, salary raise, and improvement in the opportunity for career development seems unapplicable in this context where the participants are among the senior teachers, in which the age brackets are between 40 and above 50 years. The research indicates that teachers are unique in terms of the source of motivation for individual PD, whether intrinsic or extrinsic and the aimed development. The fifth placement was “a change in profession for career advancement”. A good number of the participants are within the 40–50 years of age category where those nearing retirement indicated a lack of interest in a career change or advancement.

The finding also shows that DG32 teachers recorded higher extrinsic satisfaction as compared to DG41 teachers, with a significant value of 0.047. The former group of teachers recorded higher satisfaction because the PDP they attended served as a gateway to further education where it has been recorded as the second-highest teacher extrinsic satisfaction based on the descriptive analysis. This group of teachers is also younger than those in the DG41 group. Hence, they must have been more ambitious to continue their studies. More opportunities for PD courses could also be the reason for higher satisfaction being recorded from this group. It also shows that these teachers have accumulated new added knowledge and skills regarding their job which contribute toward the increment of self-satisfaction in teaching students at schools. Networking with teachers from other schools had tremendously helped them in sharing knowledge of related education, teaching skills, and current education issues, and it has been recorded in the descriptive analysis as the highest satisfaction that they had gained from the program.

In retrospect of the semi-structured interview, the teachers indicated a general satisfaction of the program in terms of knowledge and skills obtained, besides the lecturers' quality and qualification. This shows that the courses offered were relevant and comprehensive. The organizer of any PDP needs to look into the technological aspects to improve the content of the program as a way to ensure its relevance in catering to the current suitability and practicality to students since there is a rise in online teaching and learning during the current pandemic situation. The universal need for digital technologies requires educators to build on their existing skills where the sophisticated use of technology has taken on greater importance (Omar et al., 2018).

### Limitations of This Study

This study employed data from the Malaysian primary school ESL teachers who were doing their first-degree part-time program. The generalization of the results may be limited to this particular group of teachers. Expansion of study into other categories of groups, such as secondary school teachers, might produce more interesting findings of their satisfactions. The study had also been conducted on only thirty samplings that covered the east coast schools in Malaysia, compared to the larger areas of other states in the country, which would be more comprehensive.

### Directions for Future Research

Seminars, workshops, and future professionalism programs should be arranged in different areas, based on the target group of the teachers taking into consideration their age, seniority, and professionalism needs. Policymakers and educational administration should plan such programs as suggested by Avidov-Ungar (2016) in order to meet the dimensions of teacher PD disposition with a specific need that are suited to the different preferences and aspirations of teachers.

Identifying other characteristics that affect satisfaction should be the topic of future research. Concurrently, since PD training is a product of interest to education personnel, further efforts should be made to identify relevant attributes of potential participants and to discover the causes of continuing to participate in PD training.

## Conclusion

This research reveals the types of teacher satisfaction within a PDP. It is hoped to provide directions for policymakers and organizers of the PDP in enhancing the program by taking into consideration of teacher satisfaction, seniority, and professional background. MOE needs to organize teacher PDPs that take into consideration the quality of the program, the quality of the facilities provided, and the teaching quality of lecturers to ensure teachers benefit from it in accordance with the latter's needs. A successful program will reflect in the intrinsic and extrinsic satisfaction among the teachers, which will further produce a chain effect on their clients, who are their students, in ensuring quality education on the whole.

## Data Availability Statement

The original contributions presented in the study are included in the article/supplementary material, further inquiries can be directed to the corresponding author/s.

## Ethics Statement

The studies involving human participants were reviewed and approved by UiTM Terengganu. The patients/participants provided their written informed consent to participate in this study.

## Author Contributions

All authors listed have made a substantial, direct, and intellectual contribution to the work and approved it for publication.

## Conflict of Interest

The authors declare that the research was conducted in the absence of any commercial or financial relationships that could be construed as a potential conflict of interest.

## Publisher's Note

All claims expressed in this article are solely those of the authors and do not necessarily represent those of their affiliated organizations, or those of the publisher, the editors and the reviewers. Any product that may be evaluated in this article, or claim that may be made by its manufacturer, is not guaranteed or endorsed by the publisher.
